# Widespread non-target-site resistance in *Setaria viridis* to four classes of herbicide

**DOI:** 10.1007/s00122-026-05240-7

**Published:** 2026-05-05

**Authors:** Thomas H. Pendergast, Sailaja Maddali, Srinivasa R. Chaluvadi, Peng Qi, William K. Vencill, Jeffrey L. Bennetzen, Katrien M. Devos

**Affiliations:** 1 Institute of Plant Breeding, Genetics & Genomics, University of Georgia, Athens, GA USA; 2 Department of Plant Biology, University of Georgia, Athens, GA USA; 3 Department of Crop and Soil Sciences, University of Georgia, Athens, GA USA; 4 Department of Genetics, University of Georgia, Athens, GA USA; 5 Present Address: USDA-APHIS-BRS, Riverdale, MD USA; 6 Present Address: Goldbelt C6, 860 Greenbrier Circle, Suite 405, Chesapeake, VA USA

## Abstract

**Key message:**

Although herbicide resistance in *Setaria* is rampant and cosmopolitian across four herbicide families, we encountered little evidence of target-site resistance, indicating diverse non-target mechanisms of metabolizing, sequestering, and overwhelming herbicides.

**Abstract:**

*Setaria viridis* is a cosmopolitan weed and model genetic system with increasing reports of resistance to multiple classes of herbicides. Our goal was to assess the herbicide resistance and allelic diversity in herbicide target genes in a collection of *Setaria* genotypes from North America and Eurasia, and identify the occurrence of novel and known target-site mutations that led to resistance. A total of 214 *Setaria* genotypes were exposed to commonly used herbicides that inhibit specific genes: herbicide action class (HRAC) group 1 herbicides targeting acetyl-CoA carboxylase (ACCase), HRAC 2 targeting acetolactate synthase (ALS), HRAC 9 targeting 5-enolpyruvylshikimate-3-phosphate (EPSP) synthase, and HRAC 10 targeting glutamine synthetase. ACCase and ALS genes in 53 accessions were PCR-amplified and sequenced. Whole-genome sequencing reads covering the target genes were analyzed for an additional 98 genotypes. Herbicide trials showed that 30% of our accessions set seed following application of at least one herbicide, and 13 accessions were resistant to multiple classes of herbicides. Although there were numerous SNPs, including some known to lead to resistance, in our target genes, SNPs found predominantly in herbicide-resistant genotypes were largely intronic or synonymous. A small number of amino acid substitutions in ALS and ACCase indicated potential and incomplete resistance to HRAC 1 and 2 herbicides, but no SNPs putatively associated with herbicide resistance were identified in the other 6 target-site genes. The broader pattern of herbicide resistance in *S. viridis* is likely driven by non-target mutations that detoxify or compartmentalize applied herbicides.

**Supplementary Information:**

The online version contains supplementary material available at 10.1007/s00122-026-05240-7.

## Introduction

Green foxtail (*Setaria viridis* (L.) P. Beauv.) is one of the most widely dispersed grasses. It is present on all continents except Antarctica, ranging from the islands of Northern Canada to the Ethiopian highlands, the deserts of Saudi Arabia, and the Tasmanian forests (GBIF.org 2023). It is widely adapted to a broad range of habitats, although noticeably absent from wet tropical forest environments. Green foxtail is found mainly in disturbed habitats such as crop fields. Its general small stature and early/short developmental cycle, however, cause this weed to have a relatively low impact on the yield of associated crops (O'Donovan 1994). *S. viridis* has also become an important C_4_ photosynthesis model, given its fast life cycle, small genome size (2*n* = 18, 515 Mb), and annotated genome assemblies (Bennetzen et al. 2012; Brutnell et al. 2015; Devos et al. 2017; Doust et al. 2009; Pant et al. 2016).

Agricultural weeds, like *S. viridis,* are subjected to intense selection to resist the growing suite of commercial herbicides repeatedly and broadly applied for their control. As a result, *S. viridis* has demonstrated resistance against at least four modes of action herbicide classes (Darmency et al. 2017; Heap 2024: HRAC 1, 2, 3, 5). Weeds can escape the pressures of herbicides through two larger mechanisms, target-site and non-target-site resistance. Target-site resistance is predominantly driven by non-synonymous mutations within the herbicide target gene that decrease herbicide efficacy by altering its binding sites, but may also occur due to multiple copies or high expression of target genes (Darmency et al. 2017; Gaines et al. 2010; Sammons and Gaines 2014). For example, a Ser_264_ → Gly mutation in the *psb*A gene modifies the herbicide triazine binding site, substantially reducing the herbicide’s impact, but also decreasing photosynthetic rate because it results in inefficient electron transport in photosystem II (Arntz et al. 2000; Tian and Darmency 2006). Non-target-site resistance evolves via mutations that result in impeded absorption/translocation, vacuole sequestration, or increased metabolism of the herbicide, or localized rapid necrosis of the host (Gaines et al. 2020; Torra et al. 2021; Yuan et al. 2007). Oxidoreductase enzymes, such as cytochrome P450’s, are involved in some instances of herbicide metabolism, likely by changing herbicide hydrophobicity and binding that results in a more inert, oxidized product (Guengerich 2018; Suzukawa et al. 2021). For example, herbicide-resistant populations of *Lolium spp.* became less resistant if exposed to P450 inhibitors (Busi et al. 2017; Yanniccari et al. 2020). Understanding specific mechanisms of herbicide resistance is critical; it informs on selective pressures and agricultural best practices, and may suggest novel transgenic strategies for weed control (Darmency et al. 2017; Vencill et al. 2012).

In *S. viridis,* target-site resistance has been best documented in two classes of herbicides (as defined by the Herbicide Resistance Action Committee, or HRAC) targeting the nuclear-encoded chloroplast acetyl-CoA carboxylase (ACCase) gene (HRAC 1) and the acetolactate synthase (ALS) gene (HRAC 2). The ACCase enzyme catalyzes the initial step in lipid biosynthesis, and its inhibition rapidly leads to plant death. HRAC group 1 herbicides such as cyclohexanediones and aryloxyphenoxypropionates are largely effective only on grasses, and as such are commonly used for post-emergence grass weed control in broadleaf crops (Gronwald et al. 1992). A target-site Ile_1780_ → Leu substitution in ACCase is a well-identified mechanism of resistance that does not appear to decrease growth or reproduction (Wang et al. 2010), but at least five other mutations may confer some degree of herbicide resistance (Beckie and Tardif 2012; Darmency et al. 2017; Délye 2002). Resistance to HRAC group 1 herbicides has been identified on every continent except Antarctica in 51 grass species, including green foxtail (Heap 2024; Volenberg et al. 2002). HRAC group 2 target-site herbicide resistance is caused by mutations in the ALS gene, a crucial enzyme in the synthesis of branched amino acids (Patzoldt et al. 2005; Tranel and Wright 2002). A number of target-site mutations appear to confer resistance, with various substitutions at Pro_197_ appearing in dozens of species (Tranel et al. 2024). Over 170 plant species, including green foxtail, across all non-polar continents have reported resistance to ALS-inhibiting herbicides, making it the most documented mode-of-action resistance (Cao et al. 2023; Heap 2014, 2024).

Resistance to HRAC 9 and 10 herbicides is comparatively uncommon, purportedly due to fewer known non-detrimental target-site mutations in enolpyruvyl shikimate phosphate synthase (ESPS) and glutamine synthetase, respectively (Heap 2014; Zhang et al. 2022). However, ESPS resistance has increased in frequency recently; 60% of all reported resistance has occurred since 2010 (Heap 2024). This is likely due to the exponential increase in acreage of glyphosate application, making it the most widely used herbicide worldwide (Comont et al. 2019; Dill et al. 2008). Presently, there have been no records of *Setaria* resistance to HRAC 9 or 10 herbicides.

Here, we evaluate resistance within a diverse panel of *Setaria* to four classes of herbicide (HRAC 1, 2, 9, 10). We predict that patterns of resistance will be strongly associated with origin and phylogeny, as specific herbicide pressure varies regionally, and target-site resistance is highly heritable (Comont et al. 2019). To identify the frequency of target-site mutations, and potentially identify novel causal polymorphisms, we examined the genetic diversity of the target genes of herbicide action, and correlated nucleotide polymorphisms and heterozygosity with resistance.

## Materials and methods

### Herbicide assay

The herbicide assay panel was composed of 214 genotypes from 154 *Setaria* accessions from North America, Asia, Europe, and the Middle East, consisting of 12 *S. faberi*, 10 *S. italica*, and 192 *S. viridis* genotypes (Table S1). Here, we use “accession” to refer to a population of individuals from a specific location that could be genetically distinct or identical (Schröder et al. 2017). For 25% of accessions, multiple genotypes were analyzed, referenced herein as genotypes or lines. Fourteen *S. viridis* genotypes, identified as resistant to either HRAC 1 or 2 herbicides, were provided by Hugh Beckie (Agriculture and Agri-Food Canada). In six sequential greenhouse experiments, *Setaria* seeds were germinated in petri dishes on wet filter paper at room temperature. Single seedlings were transplanted into 6 × 6x5cm pots and grown in the UGA Plant Biology Greenhouse (33.9292446, −83.3636310) for three weeks. Three plants per genotype were exposed to a single, 4X recommended dose of five commercial herbicides in four classes: fluazifop-butyl (HRAC 1, 840 g active ingredient (ai)/hectare (ha), sethoxydim (HRAC 1, @840 g ai/ha), nicosulfuron (HRAC 2, @ 136 g ai/ha), glyphosate (HRAC 9, @1650 g ai/ha), and glufosinate-ammonium (HRAC 10, @ 1936 g ai/ha). Herbicide treatments were applied using a spray chamber calibrated to deliver 187 L ha^−1^ at 165 kPa with CO_2_ as a propellant. In each of the six trials, three individuals per accession served as controls and were misted with water, for a total of 3852 treated and untreated plants. Plants were evaluated for survival and reproduction three weeks post-herbicide exposure. Non-reproductive plants were again evaluated three weeks later for recovery and reproduction.

### Phylogenetic tree and herbicide resistance

For 152 *Setaria* lines with available SSR profiles (Schröder et al. 2017), SSR genetic distances (Dps) and Phylip v3.698 (Felsenstein 2005) were used to generate a neighbor-joining (NJ) tree. Herbicide resistance data were mapped to the NJ tree in FigTree v1.4.4 (Rambaut 2018).

### Assessing SNP variation in herbicide target genes

Target gene sequence variation was assessed for a subset of the herbicide assay genotypes by two methods: direct sequencing of ACC and ALS amplicons (53 genotypes), and analysis of available whole-genome resequencing data (98 genotypes) (Mamidi et al. 2020, Table S1). For amplicon sequencing, lines were selected from across Canada and northern USA, including lines designated as resistant to HRAC 1 or 2 (H. Beckie, *pers. comm.*); HRAC 1 resistance in *Setaria* is widespread across Canada, but was less common in the USA, likely due to differential herbicide usage (Beckie 1999; Darmency et al. 2017; www.weedscience.com). DNA was extracted from seedling leaf tissue using a modified CTAB methodology. A total of 16 and four, respectively, overlapping primer sets (Table S2) allowed PCR amplification of the entire ACCase (12.8 kb, Seita.7G030200) and ALS (1.9 kb, Seita.1G169700) genes, including at least part of the 3’ & 5’ untranslated regions (UTRs). PCR reactions (25 μl total volume) were performed using 10 ng of template DNA, 1 × Phusion High-Fidelity Buffer (NEB, USA), 0.125 μM each of forward and reverse primer, 250 μM of each dNTP, and 0.5 units (U) of Phusion High-Fidelity DNA Polymerase (NEB, USA). The PCR cycling conditions were: initial denaturation at 98 °C for 2 min, followed by 26 cycles of denaturation at 98 °C for 10s, annealing at 58 °C for 10s, and extension at 72 °C for 30s. A final extension step at 72 °C for 10 min was included. Gel-purified and barcoded amplicons for each gene were combined within an accession at equal concentrations. Libraries were constructed from pooled amplicons using the NextEra XT DNA Library Preparation Kit (Illumina) and sequenced on an Illumina NextSeq500 platform at the UGA Georgia Genomics and Bioinformatics Core (GGBC). Raw sequence reads were demultiplexed using in-house Python scripts, and adaptor sequences and barcodes were removed using “Cutadapt” (Martin 2011). Reads were aligned against the genomic sequences of the ACCase and ALS genes extracted from the *S. italica* Yugu1 v2.2 whole-genome sequence assembly (Bennetzen et al. 2012) using Bowtie2 (Langmead and Salzberg 2012).

An additional 98 *S. viridis* accessions resequenced by the Joint Genome Institute (JGI) were analyzed (Mamidi et al. 2020). Whole-genome sequence reads were aligned using Bowtie2 against the target genes plus 5 kb up- and downstream sequence, as identified in the *S. italica* v2.2. assembly in Phytozome (https://phytozome-next.jgi.doe.gov). The target gene set consisted of the ACCase and ALS gene copies amplified by PCR, as well as 5-enolpyruvylshikimate-3-phosphate (EPSP) synthase (Seita.4G023200), four homologs of glutamine synthase (Seita.1G311400, Seita.3G024100, Seita.9G118300, and Seita.9G485600), and an additional homolog of ACCase (Seita.9G271700). All genes analyzed and their location in the *S. italica* v2.2 assembly are presented in Table 1.

**Table 1:** Target genes, acronyms, their location in the *Setaria italica* v2.2 genome, and number of polymorphic loci identified by element region. Parenthetical SNPs are polymorphisms potentially associated with herbicide resistance

Gene	Acronym	Setaria italica v2.2 Gene	v2.2 Location	Gene size (bp)	# UTR SNPs	# Intronic SNPs	# Exonic SNPs	# Candidate Substitutions^1^
Acetyl-CoA carboxylase	ACCase7	Seita.7G030200	scaffold_7:8,711,297. 8,726,543 reverse	15,246	118 (24)	321 (24)	218 (8)	5
Acetyl-CoA carboxylase	ACCase9	Seita.9G271700	scaffold_9:23,428,599. 23,440,836 reverse	12,237	7 (2)	79 (35)	17 (4)	0
Acetolactate synthase	ALS	Seita.1G169700	scaffold_1:24,269,905. 24,272,717 forward	2812	41 (8)	0	166 (9)	3
5-enolpyruvylshikimate-3-phosphate	EPSP	Seita.4G023200	scaffold_4:1,557,888. 1,561,075 forward	3187	0	66	5	0
Glutamine synthetase	GlutSynth1	Seita.1G311400	scaffold_1:37,311,880. 37,315,284 reverse	3404	3 (0)	51 (2)	5 (0)	0
Glutamine synthetase	GlutSynth3	Seita.3G024100	scaffold_3:1,455,306. 1,459,782 reverse	4476	22 (6)	40 (16)	11 (6)	0
Glutamine synthetase	GlutSynth9_1	Seita.9G118300	scaffold_9:7,327,714. 7,330,961 forward	3247	4 (2)	68 (19)	0	0
Glutamine synthetase	GlutSynth9_2	Seita.9G485600	scaffold_9:52,568,416. 52,571,465 forward	3049	4 (0)	8 (6)	0	0

^1^Candidate substitutions are exonic SNPs identified as potentially associated with herbicide resistance that result in amino acid substitution

Nucleotide polymorphisms (NPs, referring to both single nucleotide polymorphisms and indels) from amplicon and whole-genome sequences were jointly called using the Genome Analysis Toolkit (GATK) version 3.4, UnifiedGenotyper function (McKenna et al. 2010). We removed NPs with three or more alleles and a quality depth value < 8. Allelic status of each locus was determined as in Qi et al. (2018).

### Determining associations between NPs and resistance

For each herbicide and corresponding target gene, *Setaria* accessions were coded as resistant or susceptible. Conservatively, if any of the three replicate individuals reproduced or displayed active growth six weeks following the 4X herbicide application, the line was designated as resistant. At each identified polymorphic locus, we asked if resistant lines had different allele frequencies, including heterozygosity, relative to susceptible lines. One-sided Fisher exact tests were performed at each locus to identify significantly different allele frequencies between resistant and susceptible genotypes. This proved too conservative, so we also identified any locus with at least l5% allelic frequency difference between the resistant and susceptible. All flagged loci were determined to be UTR, intronic, or exonic; exonic NPs were examined whether they led to an amino acid change. Any such polymorphism was considered a potential causal herbicide resistance substitution. We then compared amino acid substitutions resulting from NPs to published amino acid changes putatively associated with herbicide resistance (Beckie and Tardif 2012; Heap 2024; Zhang et al. 2022). To test the hypothesis that heterozygosity may influence resistance, we conducted Chi-square tests for homozygosity predictions for resistant *versus* susceptible lines for the 10 appropriate herbicide/target gene pairs.

## Results

### Herbicide resistance

**Fig. 1:**
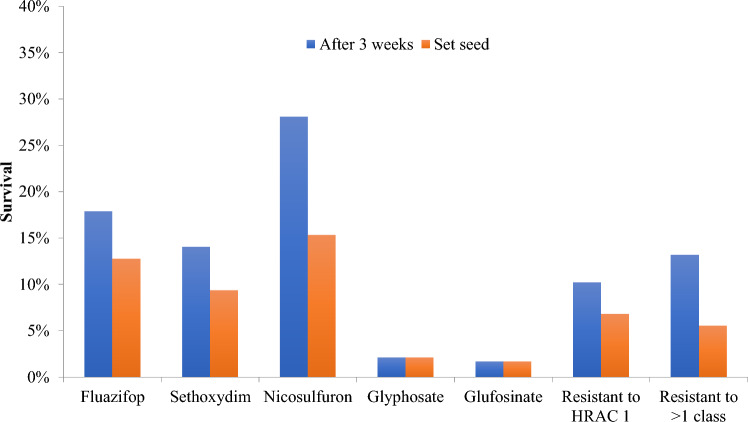
Survival of 213 *Setaria* genotypes after exposure to 4X recommended concentrations of fluazifop, sethoxydim, nicosulfuron, glyphosate, and glufosinate

**Fig. 2:**
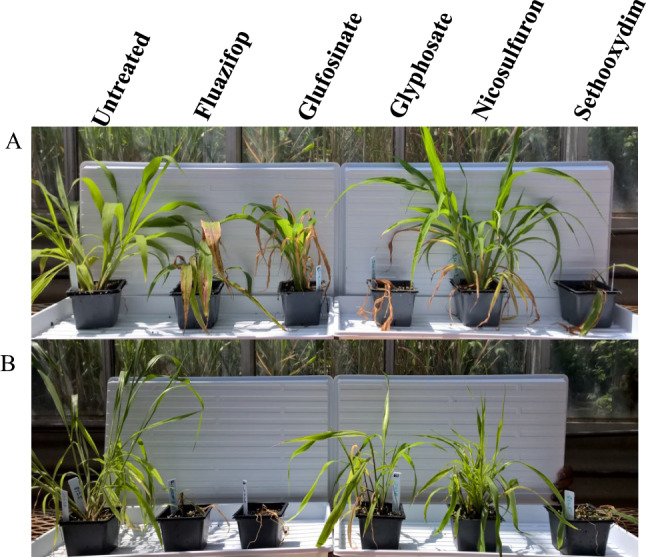
Response of Ise-1719 (**A**) and 8125 (**B**) *S. viridis* genotypes to five herbicides

Broadly, herbicide resistance was widespread, with 40% of all genotypes resistant to one or more herbicides (Fig. 1, Table S3). Resistance to HRAC 1 and HRAC 2 was the most common, with 18%, 14%, and 28% of *Setaria* lines having at least one replicate (of three) surviving exposure to fluazifop-butyl (HRAC 1), sethoxydim (HRAC 1), and nicosulfuron (HRAC 2), respectively. The resistance response to the two HRAC 1 herbicides was similar but not identical; only 10% of genotypes were resistant to both fluazifop-butyl and sethoxydim. Not all lines that survived at three weeks were able to reproduce. Only 9–13% of lines exposed to HRAC 1 herbicides and 15% of lines exposed to nicosulfuron (HRAC 2) set seed. Resistance to glyphosate and glufosinate was rare, with only 2% of genotypes surviving exposure and reproducing. Thirteen percent of genotypes exhibited some resistance to multiple HRAC classes of herbicide, mostly HRAC 1 and HRAC 2 (Fig. 1, 2). However, a minority of genotypes had other combinations of herbicide resistance. For example, accession 8125 had some resistance to both glyphosate and nicosulfuron, Ise-1719 had resistance to glufosinate and nicosulfuron, and Ise-1430 had resistance to glufosinate and fluazifop (Table S3).

Herbicide resistance was observed in genotypes from every continent sampled (particularly to nicosulfuron), while 40% of *Setaria* lines from the USA were resistant to at least one herbicide (Table 2). Resistance to HRAC 1 and 2 occurred in 15–21% of USA lines, and a single genotype each was resistant to glyphosate (1211-P1) and glufosinate (1181-P2). Canadian and East Asian accessions had intermediate HRAC1 and 2 resistance (13–26%), and a few lines were resistant to glufosinate. It should be noted, however, that 38% of the Canadian lines analyzed were preselected because of their known resistance to at least one HRAC1 or 2 herbicide (Table S3). Accessions from South Asia and the Middle East had high frequencies of resistance to nicosulfuron, moderate HRAC1 resistance, and a few lines resistant to glyphosate. All European lines were susceptible to all herbicides, except for one genotype that set seed after exposure to nicosulfuron.

**Table 2:** Frequency of herbicide resistance by world regions, as determined by herbicide assays

	Origin	Fluazifop	Sethoxydim	Nicosulfuron	Glyphosate	Glufosinate	% resistant	Total Lines
Reproduction after exposure	USA	20.8%	15.6%	17.7%	1.0%	1.0%	39.6%	96
	Canada	10.8%	10.8%	5.4%	0%	2.7%	13.5%	37
	East Asia	0%	3.7%	14.8%	0%	7.4%	22.2%	27
	South Asia	0%	0%	35.7%	7.1%	0%	35.7%	14
	Middle East	15.0%	0%	20.0%	10.0%	0%	40.0%	20
	Europe	0%	0%	5.3%	0%	0%	5.3%	19
Survival 3 weeks after exposure	USA	25.0%	21.9%	34.4%	1.0%	1.0%	50.0%	96
	Canada	13.5%	13.5%	13.5%	0%	2.7%	24.3%	37
	East Asia	7.4%	18.5%	25.9%	0%	7.4%	37.0%	27
	South Asia	21.4%	7.1%	42.9%	7.1%	0%	50.0%	15
	Middle East	20.0%	0%	45.0%	10.0%	0%	55.0%	20
	Europe	0%	0%	15.8%	0%	0%	15.8%	19

**Fig. 3:**
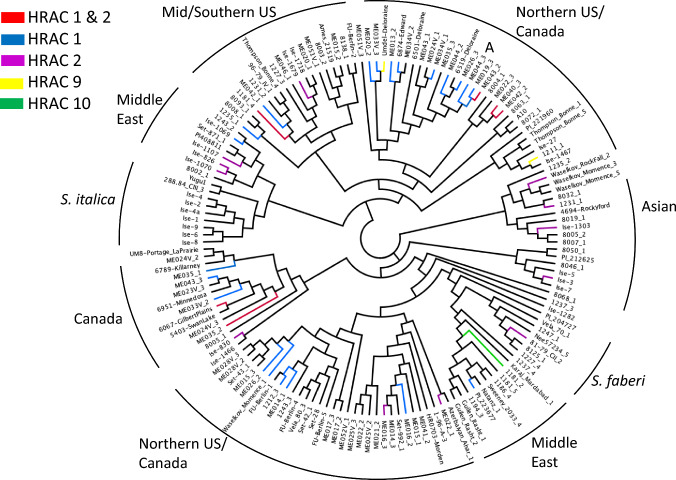
Neighbor-joining tree for 152 *Setaria* lines, using SSR genetic distances (Dps). Accessions are color coded according to resistance to HRAC herbicide families; black is susceptible. *Setaria* lines are identified in Table S1. “A” is a Canadian resistant clade

Herbicide resistance is found throughout the *Setaria* dendrogram (Fig. 3). The vast majority of sub-clades contain resistance to HRAC 1, 2, or both, but sister lineages are frequently susceptible. An exception to this pattern is a highly resistant clade comprised of six Canadian *S. viridis* lines (Fig. 3, “A”) with four genotypes being resistant to HRAC1 (ME19, ME26, ME44-1, and ME44-2) and ME43 being resistant to both HRAC 1 and 2. In contrast, 6519-Deloraine, well within this clade, is susceptible to all herbicides.

### Assessing nucleotide variation in herbicide target genes

We identified 4815 polymorphic loci across eight potential target genes (Table 1). However, most recorded NPs (74%) were located in the UTR and adjacent intergenic regions, and are maintained here for alignment purposes. A total of 3550 NPs occurred intergenically at an average density of 1.9 NPs per 100 bp, though this varied by target gene, from 0.66 per 100 bp for scaffold 9 ACCase to 5 per 100 bp for ALS. Most (74%) intragenic NPs were located within introns. Nucleotide polymorphism data are presented in Tables 1, S4.

### Determining associations between NPs and resistance

We found few and weak associations between coding region variants leading to amino acid changes and herbicide resistance. The Fisher exact test for allelic frequency differences between resistance and susceptible lines proved conservative, finding 36 significant loci across all genes (10 intronic NPs in ACCase 7 w/fluazifop-butyl, 11 exonic and 11 intronic NPs in ACCase7 w/ sethoxydim; 3 exonic NPs in ALS, 1 intronic NP in GlutSynth9_2). This low association rate is likely due to the substantially lower numbers of resistant lines, relative to susceptible, making traditional statistics challenging (Ryman et al. 2006). The blanket 15% allelic frequency difference, in contrast, identified 171 intragenic NPs for investigation, and all NPs found by the Fisher exact test (Table 1). The vast majority of potential causal NPs were intronic or synonymous. Although we encountered two known resistance mutations, both for HRAC1, in ACCase7, these were not conclusively associated with resistance in our study (Table 3). Of the four genotypes containing the ACCase resistance substitution Asp_2078_ → Gly (Beckie and Tardif 2012), only two genotypes (ME35-P2 & 6067) were resistant to fluazifop-butyl and sethoxydim; genotypes Set 28 and 5403 also carried this allele but neither was resistant to either HRAC 1 herbicide at the 4X dose used in our experiments. Similarly, two genotypes (ME26_P2, ME26_P3) contained the known resistance allele ACCase Gly_2096_ → Ala (Beckie and Tardif 2012), potentially explaining the resistance of both genotypes to sethoxydim, but only ME26_P2 was resistant to fluazifop-butyl. A total of 19 and 12 genotypes without Asp_2078_ → Gly or Gly_2096_ → Ala were resistant to fluazifop-butyl and sethoxydim, respectively.

**Table 3:** Target-site mutations identified in our Setaria panel with identified or putative association for fluazifop-butyl and sethoxydim resistance. Parenthetical "B," "H," and "C" indicate homozygous alternate allele, heterozygous, or potentially either, respectively

Herbicide	HRAC Group	Target Gene	# Resistant: Susceptible Accessions	Published/Putative Resistance SNPs	Resistant Accessions with Substitution	Susceptible Accessions with Substitution	% of Resistant Accessions with SNP	% of Susceptible Accessions with SNP
Fluazifop-butyl	1	Acetyl-CoA carboxylase 7	23:73	Val_1523 →_ Ile	8B, 5H, 2C^1^	24B, 11H, 5C^1^	83.3%	60.6%
				Asp_2078_ → Gly^2^	6067(H), ME35-P2(C)	Set 28(H), 5403(B)	10.5%	2.8%
				Gly_2096_ → Ala^2^	ME26_P3(B)	ME26_P2(B)	5.0%	1.4%
Sethoxydim	1	Acetyl-CoA carboxylase 7	18:78	Ile_978_ → Val	1212-P3(H), 6067(H), ME35-P2 (H)	ME35-P3(B)	21.4%	1.4%
				Lys_1005_ → Glu	1212-P3(H), 6067(H), ME35-P2 (H)	ME35-P1(H), ME35-P3(B)	21.4%	5.5%
				Ala_1214_ → Val^3^	1212-P3(H), 6067(B), ME35-P2(H)	ME35-P3 (C)	20%	1.4%
				Gly_1221_ → Ala^3^	1212-P3(H), ME35-P2 (C), 6067(B), ME42-P1(H)	SET 992(H)	25%	1.4%
				Val_1523 →_ Ile^3^	8B,4H, 1C^1^	24B, 12H, 6C^1^	92.9%	64.7%
				Asp_2078_ → Gly^2^	6067(H), ME35-P2 (C)	Set 28(H), 5403(B)	13.3%	2.7%
				Gly_2096_ → Ala^2,3^	ME26_P2(B), ME26_P3(B)	-	12.5%	0%
Nicosulfuron	2	Acetolactate synthase	13:83	Ala_52_ → Pro, Val, Leu	1231-P1(B), ME35-P2 (H), ME43-P2(H)	4B, 4H^1^	38%	12.1%
				His_243_ → Pro	ME16-P3(H), ME35-P2(H), 6067(H)	6H^1^	25.0%	7.7%
				Arg_291_ → Cys^2,3^	ME35-P2(H), 6067(H)	-	16.7%	0%

^1^Allele frequencies for putative resistance SNP. Genotype identity available in Table S3

^2^Resistance substitutions known from the literature

^3^Putative SNPs also identified via Fisher exact test

However, we did encounter a series of other NP coding for amino acid substitutions in ACCase7 that were more common in herbicide-resistant relative to susceptible lines (Table 3). Substitutions Ile_978_ → Val, Lys_1005_ → Glu, Ala_1214_ → Val, Gly_1221_ → Ala occurred between 21 and 27% in fluazifop-butyl resistant lines, and rarely (1.4%-5.5%) among the susceptible. Three genotypes (1212-P3, 6067, and ME35-P2) had at least three of these polymorphisms. Additionally, the overwhelming majority of fluazifop and sethoxydim resistant lines (82.4% and 92.9%, respectively) contained a Val_1523_ → Ile substitution, while this polymorphism was found in just over half of susceptible lines.

Three novel acetolactate synthase substitutions were overrepresented in nicosulfuron resistant lines (Table 3). The substitutions Ala_52_ → Pro, Val, Leu were found in 38% of resistant lines (compared to 23% in susceptible), and the substitutions His_243_ → Pro and Arg_291_ → Cys were, respectively, 2.8 and 5.5 times more prevalent among resistant lines. Genotype ME35-P2 held the first two substitutions; genotype 6067 had all three.

Although target-site mutations have been identified for glufosinate (Zhang et al. 2022) and glyphosate (Nandula et al. 2013; Wakelin and Preston 2006), we did not find these polymorphisms in our panel. Indeed, excepting the two known ACCase mutations effective in providing HRAC 1 resistance noted above, we found no other published herbicide resistance polymorphism in ACCase on scaffold 9 (ACCase9), ALS, glutamine synthetase or EPSP synthase, or their homologs.

We found little support for the hypothesis that higher rates of heterozygosity across the target gene may affect resistance. For ACCase9, EPSP synthase, and all homologs of glutamine synthetase, heterozygosity was low, averaging 0.3% of loci (Table S5). ALS and ACCase7 had higher rates of heterozygosity, with 5% and 7%, respectively. Although Chi-squared tests found significant differences in allelic frequency between susceptible and resistant genotypes for four herbicide/gene combinations (nicosulfuron-ALS, fluazifop-ACCase7, sethoxydim-ACCase7, sethoxydim-ACCase9; see Table S5), this was mostly driven by the large number of loci analyzed; allelic frequencies at polymorphic loci across the gene generally varied less than 5% between susceptible and resistant lines. An exception to this pattern was for ACCase9 when exposed to sethoxydim; resistant genotypes were 1.73 times more likely to have the alternate B allele across the gene than susceptible genotypes (Table S5). Heterozygosity was also higher in resistant compared to susceptible lines, though levels remained below 1% (0.8% vs 0.1%, respectively).

## Discussion

Our aim was to determine cosmopolitan patterns of resistance to four common herbicide families in a diverse *Setaria* panel, and then establish associations between resistance and both known and novel nucleotide polymorphisms in target genes. Although we found widespread herbicide resistance, including the first documented resistance in *S. viridis* to glyphosate or glufosinate, a few instances of multiple herbicide family resistance, and extensive target gene diversity, we were unable to confirm causality of any prevalent target-site mutation to resistance observed under our experimental conditions (4X recommended herbicide concentration). Although the literature is rife with specific mutations that interfere with herbicide binding sites (Heap 2024), our results beg the following questions: Can resistance evolve faster, and is it more effective and against a wider range of herbicides, through non-target-site mechanisms?

### Herbicide resistance is pervasive and overdispersed

Almost 40% of our broad panel had some resistance to at least one of the tested herbicides, indicating the intense selective pressure of modern agriculture (Baucom 2019). We encountered resistance to every class and herbicide tested, including glyphosate and glufosinate (Figs. 1, 2). While there was significant overlap in resistance of genotypes to the two HRAC 1 herbicides tested (fluazifop-butyl, sethoxydim), survival to one HRAC 1 herbicide did not guarantee survival to the other. Importantly, 13% of tested lines survived multiple families of herbicide, though only 5.5% were able to reproduce subsequent to herbicide treatment. The vast majority of multiple family resistances were for HRAC 1 and 2, likely due to the intense and cosmopolitan use of these herbicides. For example, all replicates of 6067-Gilbert Plains MB Canada set seed after being exposed to fluazifop-butyl, sethoxydim, and nicosulfuron. Multiclass herbicide resistance has been documented particularly in *Lolium rigidum*, a noxious weed that is widespread across much of Europe and Australia. While multiple target-site mutations have been identified in *Lolium* for resistance to ACCase and ALS (Anthimidou et al. 2020), non-target-site diversity also represents a driving evolutionary force in this species*,* providing a generalist approach to a herbicide mosaic across the landscape (Torra et al. 2021; Yu and Powles 2014).

We found a much stronger effect of *Setaria* accession country of origin than phylogeny on resistance (Table 2, Fig. 3). This pattern is not necessarily surprising; rates of selection against variable herbicide application practices likely operate on much faster time frames than drift, especially in an inbreeding system. Phylogenetically, *Setaria* herbicide resistance is overdispersed, appearing as largely independent evolutionary events, rather than forming distinct resistant lineages. Almost every major *Setaria* clade had at least one resistant line, though resistant sister species were uncommon. An exception to this was a Canadian clade (“A,” Fig. 3), where only *S. viridis* line 6519 was susceptible; the other five lines in the clade were resistant to HRAC 1 herbicides with line ME43 being resistant to both HRAC 1 and 2. While Occam’s Razor suggests a single resistance origin and subsequent loss in 6519, the pattern of overdispersion across the phylogeny may also predict multiple independent events and potentially different causal mutations. Alternatively, 6519 may be resistant to herbicide concentrations lower than used in this experiment; this accession was previously indicated as resistant to HRAC 1 (H. Beckie, *pers. comm.*).

In general, USA and Canadian *S. viridis* accessions exhibited similar patterns of herbicide resistance, though the occurrence was substantially lower in Canadian lines. The vast majority of North American accessions were collected in roadsides adjacent to active agricultural fields (M. Estep, *pers. comm.*). It is very likely that these lines have been exposed to recurrent bouts of herbicide selection that may explain the high rates of resistance in the USA. All tested regions had genotypes that had some resistance to HRAC 2, though South Asian and Middle Eastern lines had significantly higher rates of resistance, potentially indicating higher usages of HRAC 2 herbicides, a reliance on a single class of herbicide, and potentially lower application rates (Montull and Torra 2023). Conversely, low occurrence rates of nicosulfuron resistance in Europe may be due to a greater reliance on crop and herbicide rotation, though resistance to ALS- and ACCase-inhibiting herbicides is increasing rapidly (Montull and Torra 2023). Glyphosate and glufosinate resistance were absent or exceedingly rare across continents. Given the overdispersed nature of *Setaria* resistance, these patterns predict resistance as a derived trait dependent on the herbicide practices of the region. It should be noted that genotype origin in our panel is strongly unbalanced; USA genotypes were overrepresented in the panel (46%), and geographic patterns of resistance here may be attributed to selection bias.

### Ubiquitous non-target-site herbicide resistance in Setaria

Our inability to identify causal target-site mutations in a diverse panel is in stark contrast to the long-standing idea that most herbicide resistance is due to selection for mode-of-action substitutions (Powles et al. 1996). In fact, we identified multiple genotypes with known target-site resistance mutations, which did not confer herbicide resistance in our study. Although two genotypes with known ACCase Asp_2078_ → Gly substitutions were resistant to both ACCase inhibitor herbicides, two other genotypes (Set 28 and 5403) were susceptible to both HRAC 1 herbicides tested, despite having the same polymorphism. Similarly, an ACCase Gly_2096_ → Ala target-site mutation potentially insulated genotypes ME26_P2 and ME26_P3 from fluazifop-butyl, but only ME26_P3 from sethoxydim. An additional 19 and 12 genotypes without known target-site substitutions were resistant to fluazifop-butyl and sethoxydim, respectively.

We did find a series of novel ACCase7 and ALS polymorphisms that were overrepresented in resistant lines (Table 3), though these also seem far from a smoking gun of herbicide resistance. Four previously unreported substitutions in ACCase7 (Ile_978_ → Val, Lys_1005_ → Glu, Ala_1214_ → Val, Gly_1221_ → Ala, the latter two also indicated by Fisher exact test) appeared potentially associated with sethoxydim resistance, though these were potentially explanatory for a small number of resistant lines and four *Setaria* genotypes with one or two of these mutations were also susceptible. Three resistant Northern US/Canadian, but largely unrelated, genotypes (Fig. 3) held three or more substitutions, and one genotype (6067) held all but one identified amino acid changes here. Similarly, three substitutions in ALS were more prevalent in resistant lines, but an equal to greater number of genotypes with said mutations were susceptible even with two of the mutations.

Given there is no easily identifiable target-site mutation(s) for resistance in ACCase7 or ALS in our study, we may conclude that non-target mechanisms are operating, as the data also suggest for other examined genes. An alternative interpretation is that a mélange of resistance mechanisms exist for HRAC 1 and 2 herbicides in this panel. Any single, above-mentioned substitution may be a causal site of resistance, but is only marginally effective at our herbicide concentrations, allowing *Setaria* lines to occasionally recover and even reproduce. Plants may also be able to oversaturate target-site effects by increasing target gene copy number (Jugulam et al. 2014) or overexpressing the target gene (Brunharo et al. 2019; Gaines et al. 2010). Indeed, Dong and colleagues (2011) drastically increased maize sethoxydim resistance by overexpressing an ACCase allele from a resistant *S. italica* line. There may also be a mutation dosage effect, where individual mutations may not convey consistent resistance, but genotypes carrying multiple alternative alleles are increasingly resistant. This is an emerging potential mechanism (Murphy and Tranel 2019), and has been demonstrated with glyphosate resistance where two or three target-site mutations in EPSP synthase result in greater resistance than a single amino acid substitution (Heap and Duke 2018; Perotti et al. 2019). Here, genotype 6067, which is resistant to fluazifop, sethoxydim, and nicosulfuron, held all identified substitutions in ALS and ACCase7, except Gly_2096_ → Ala (Table 3). Generally, it should also be noted that increasing amino acid substitutions in vital and conserved genes is predicted to decrease overall viability. However, while the above scenarios may be operating, this is only a potential explanation for our HRAC 1 and 2 herbicides and scaffold 7 acetyl-CoA carboxylase. We found no other potentially causal mutations in the six other target-site genes with glufosinate and glyphosate.

Our findings do not necessarily counter well-established observations of target-site mutations conferring herbicide resistance in *Setaria* (Cao et al. 2023; De Prado et al. 2004; Délye 2002; Laplante et al. 2009; e.g., Marles et al. 1993; Shukla et al. 1997; Wang et al. 2010). However, the pervasive dearth of apparent target-site variants consistently associated with resistance in our study does indicate that non-target-site mechanisms are likely operating in agricultural settings (Yuan et al. 2007). There are a number of non-target mechanisms by which plants can escape or ameliorate the deleterious effects of herbicides (Darmency et al. 2017; reviewed in Délye et al. 2013; Gaines et al. 2020; Ghanizadeh and Harrington 2017). Plants may be able to impede herbicide uptake via leaf cuticle properties or altered intracellular or cross-tissue transport. Sequestration or metabolism of pesticide compounds through biochemical degradation can decrease herbicide effectiveness. For example, nicosulfuron resistance in *Setaria faberi*, among others (Busi et al. 2017; Guengerich 2018; Suzukawa et al. 2021), can be reversed by cytochrome P450 inhibitor application, indicating that P450 interactions are buffering herbicide activity (Papapanagiotou et al. 2025).

Although all herbicide resistance mechanisms previously reported in *S. viridis* are attributed to target-site variation (Darmency et al. 2017; De Prado et al. 2004), non-target-site resistance mechanisms for ALS- and ACCase-inhibiting herbicides have been identified in a number of species (Ghanizadeh and Harrington 2017). Torra et al. (2021) found increased production of Glutathione-S-transferase (GST) enzymes in herbicide-resistant *Lolium rigidum* populations; GSTs catalyze the binding of glutathione to specific herbicide compounds, rendering the herbicide ineffective (Dixon et al. 1998; Ghanizadeh and Harrington 2017). Similarly, Cytochrome P-450 enzymes can facilitate oxidation of herbicides, altering their intracellular properties and interfering with transport, greatly limiting their deleterious effects (Yuan et al. 2007). These processes can be effective against multiple herbicides and herbicide classes, and are the likely mechanisms for cross-family herbicide resistance. As opposed to the immediate effects of single substitutions in target-site resistance, the evolution of non-target-site resistance may increase over time and herbicide events, potentially due to stacking of related metabolism and/or interference alleles (Gressel 2009). Here, it is also possible that stacked non-target AND marginally effective target gene mutations can result in potent resistance.

One caveat to our study is that our herbicide application design may provide different resistance metrics than other controlled tests or agricultural use (Barbieri et al. 2022; Cao et al. 2023; Desai et al. 2025; Sutherland et al. 2025). Our design rationale was that regrowth and reproduction following exposure to herbicides at four times recommended levels would indicate truly “resistant” genotypes. We concede that use of alternative exposures may lead to different resistance patterns, though our concentrations are well within many dose–response experiments (Cao et al. 2023; Depetris et al. 2024; Huang et al. 2021; Nelson et al. 2025). To that point, Hugh Beckie provided Canadian *S. viridis* seed from fifteen HRAC 1, 2, and 3 “herbicide-resistant populations,” though the germplasm was acknowledged to be a mix of resistant and susceptible genotypes. In our tests, ten of these accessions showed 100% mortality to all herbicides. Three accessions (HR0703, 2643, 6874) had extensive resistance (88% reproduction) to both fluazifop-butyl and sethoxydim, and all replicates of accession 6067 reproduced after exposure to fluazifop-butyl, sethoxydim, and nicosulfuron. Interestingly, a majority of UMDEL-Deloraine, an accession purportedly resistant to HRAC 3, reproduced after exposure to glyphosate (HRAC 9). Increased herbicide dosage is generally thought to overwhelm non-target-site mechanisms of herbicide resistance (Gardner et al. 1998), but high dosages may overcome target-site mutations as well.

## Conclusion

Although we found rampant resistance across four herbicide families, hundreds of polymorphic intragenic loci in target genes, and several known resistance target-site mutations in our target genes, we encountered few, if any, instances of target-site resistance in a diverse *Setaria* panel under our experimental conditions. Indeed, the identification of target-site mutations may no longer be the smoking gun of herbicide resistance mechanisms. The complex and evolving non-target mechanisms of impeding, metabolizing, sequestering, and otherwise overwhelming applied herbicides may be an incremental and broad strategy for plants to manage a shifting mosaic of herbicides, without the fitness costs associated with amino acid substitutions in crucial metabolic genes.

## Supplementary Information

Below is the link to the electronic supplementary material.

Supplementary file1 (XLSX 1625 KB)
